# Is the push-pull paradigm useful to explain rural-urban migration? A case study in Uttarakhand, India

**DOI:** 10.1371/journal.pone.0214511

**Published:** 2019-04-02

**Authors:** Ellen M. Hoffmann, Verena Konerding, Sunil Nautiyal, Andreas Buerkert

**Affiliations:** 1 Organic Plant Production and Agroecosystems Research in the Tropics and Subtropics, Organic Agricultural Sciences, Universität Kassel, Witzenhausen, Germany; 2 Centre for Ecological Economics and Natural Resources, Institute for Social and Economic Change (ISEC), Nagarabhavi, Bangalore, India; US Geological Survey, UNITED STATES

## Abstract

The present study explored the motivation of rural-urban migrants who moved from the Himalaya foothills of Uttarakhand to its capital city, Dehradun. A survey of 100 migrant families reported their socio-economic profile before and after migration, personal and general reasons for migration, problems in the village and in the city, and perception of push- and pull factors. A remote sensing-based analysis of land cover and forest changes was conducted for two villages of the migrants’ origin, aiming to link the reasons for migration to land cover changes. This was contextualised by reported large scale changes in forest cover. Major reasons for migration mentioned in this study were education, employment opportunities with the associated income, and facilities. These were perceived as both, push and pull factors, whereas environmental factors ranked very low. Declining environment or agriculture were never mentioned spontaneously as personal reason, and only occasionally as a presumed general reason for migration, but were frequently confirmed as a major problem in the village. Thus, although such problems existed, they seemed not a major driver of rural-urban migration. For most of the respondents their migration resulted in a profound change of livelihoods and significantly improved their socio-economic situation. Land and forest cover around the chosen villages fluctuated by up to 15% with a trend to increasing forest cover in recent years. At the district and state scales, forest cover was rather stable. These results question the narrative of deforestation and environmental degradation in the Himalayas as major push-factors for rural-urban migration in Uttarakhand. Even if environmental constraints were felt, it was rather the differences in socio-economic opportunities (education, employment, facilities) that drove people to migrate to the city. Regarding the push-pull paradigm, we conclude that scenarios of external conditions under which people migrate cannot be evaluated without taking the migrants’ attitudes and choices into account.

## Introduction

Urbanization is a global phenomenon with major implications for people and the environment [1, 2]. Only 20% of the countries with an average annual per capita income of US $ 1,000 to 2,000 were significantly urbanized in 1960; in 2016, this number had risen to over 50%. It is estimated that by 2030, the gross number of the urban population in developing countries will have doubled compared to 2005, while the extent of built-up urban areas may even triple [3, 4]. Besides its demographic impact, large-scale, rural-urban migration affects both the patterns of urban growth at the destination and land cover and land use in the region of the migrants’ origin. With respect to agriculture, urban sprawl has been shown to take prime agricultural lands out of production [5], while in abandoned rural regions agricultural land is left uncultivated [6, 7]. To date, few studies [2, 8, 9] have addressed the trade-offs between losses in agricultural production, changes in the livelihoods of (former) farmers, and gains in regulatory functions of natural habitats in response to rural-urban migration in an integrated approach.

**Fig 1:**
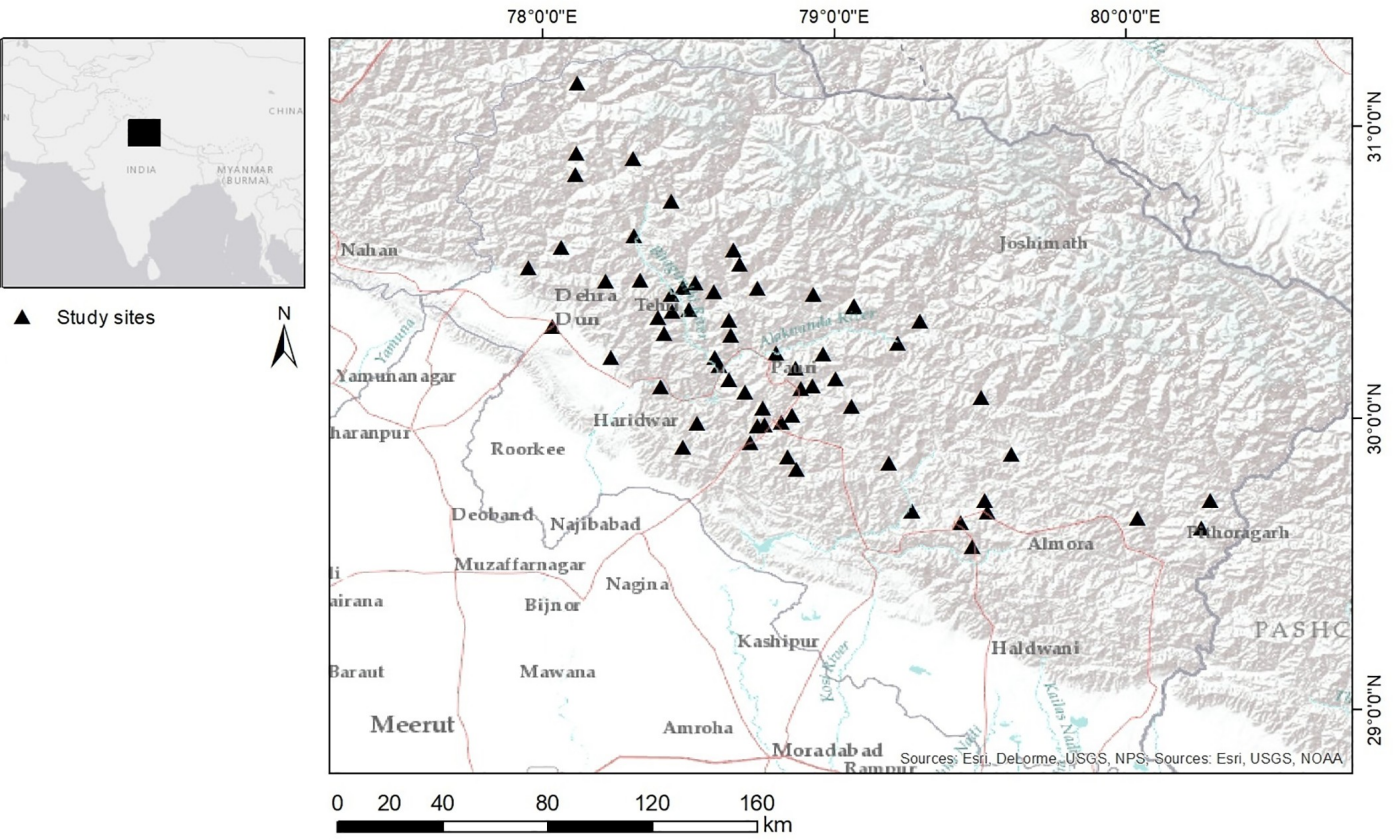
Location map of the study area. Uttarakhand in Northern India; triangles mark the migrants’ villages of origin. (Sources: Esri, DeLorme, USGS, NPS; Esri, USGS, NOAA).

The Central Himalayan foothills in Northern India (Fig 1) are a region characterized by marginal agricultural productivity, widespread rural poverty [10, 11, 12, 13] and high vulnerability to natural disasters [7, 12, 14]. The region also has experienced significant rural out-migration in recent decades [13, 15]. Despite its low productivity [7, 10, 13], agriculture is still the prime source of livelihood for 65% of the population [14, 16] (80% in 2007 according to [12]). Over the last decades local incidents of landslides, flash floods, earthquakes, and forest fires were recorded in Uttarakhand almost every year, and some major disasters struck the state in the late 1990s and between 2010–2013 [14]. Deforestation, human interventions for development projects, and climate change were held responsible for this in the scientific and public discourse [7, 14, 17]. Urbanisation in Uttrarakhand, with only six cities above 100,000 inhabitants, was reported at 30.55% in 2011 [16, 18]. In India, rural-urban migration accounts for ca. 20% of the gross internal migration flows [19, 20, 21], and between 1961 and 2011, rural-urban migration contributed 19 to 22% to urban growth [21]. In Uttarakhand, as in the majority of other Indian states, 80% of the migration flows were intra-state [20]. Dehradun has grown by 20% from 2000 to 2010, both in terms of population and built-up housing area [22]. It can thus be assumed that the city has attracted numerous migrants from the surrounding rural districts.

In the present study, Uttarakhand was chosen as an example of an Indian state where substantial rural-urban migration from the hilly, remote rural areas to the regional capital Dehradun has been documented [13, 15], and where rural poverty and environmental degradation were often discussed as major driving forces [7, 14, 15]. The motivation for rural-urban migrants has often been analysed under the (rural) push *versus* (urban) pull theory [23–27]. Rural push factors include poverty, inequitable land distribution, environmental degradation, high vulnerability to natural disasters, and violent conflicts while urban pull factors include better employment and education opportunities, higher income, diverse services, and less social discrimination in the cities [28–31]. If environmental degradation represents “the main (if not the only)” reason for migration, migrants were considered as “environmental refugees”, thus exemplifying a case of straightforward push dynamics [32, 33].

Following these lines of thought the main hypothesis of the present study was that ‘*People of rural Himalayan Uttarakhand are pushed to migrate to the city due to the deterioration of their environmental resource base*.’

The corresponding guiding questions were:

- Is rural-urban migration in Uttarakhand motivated by push- or pull-factors?
- Are environmental factors a major driver of the migration?
- Does migration have a positive or negative effect on the life of the migrants in the destination city?
- Does migration have a positive or negative effect on land cover and environment in the rural areas of origin?

The study combined a survey of 100 migrant households in Dehradun with a GIS-based land use analysis to verify environmental changes in some villages of origin, and an assessment of changes in forest cover at larger scales, based on Indian government statistics.

## Material and methods

### The survey

As outlined in the introduction, **Uttarakhand**, located in the Himalayan foothills between 30° 15' N and 79° 15' E at elevations between 1500–7816 meters (Fig 1), represents a region with many apparently typical push-factors for migration, while **Dehradun,** the state’s capital and biggest city (575,000 inhabitants [16]) has many features of a typical urban-pull attractor.

The survey sample comprised 100 migrant households with a total of 437 members visited in 24 neighborhoods in the city of Dehradun. By semi-structured interviews, information was collected about the village of origin, household characteristics, the migrants’ socio-economic status, their motivation for migration, and their perceived and actual outcome (self-assessed status). The questionnaire was designed to dissociate the personal reasons for migrating from common narratives, and to question and re-evaluate the perception of push and pull factors of migration. It asked open questions about the *personal reasons* for migrating on the one hand, and the respondent’s opinion about the *general reasons* for rural-urban migration on the other hand. It then inquired about households’ problems before and after migration, offering a list of potential answers with a yes/no choice, while allowing further explanations. The list was the same for village- and city-related problems. Last, respondents were asked about their personal sense of being pushed from the village or pulled to the city, and about which push or pull factors were considered the most relevant. This allowed a differentiated interpretation of household’s reasons to move.

Prior to application, the survey was broadly discussed with two independent sociologists in India who deemed that it met the ethical and cultural requirements applying to social research in the study area in Northern India. The type of questions asked in the household survey did not collect information that would identify human subjects or private information, and was fully compliant with the national and local norms, values and traditions. Subsequently, the questionnaire was tested with a few households and particular care was taken to examine whether any of the questions had the potential to upset the interviewees, which was determined not to be the case. The respondents were assured that participation in the survey was anonymous, voluntary, not related to any government actions, and no benefits were associated to their decision to participate or not. Verbal consent was taken in privacy within the family, to avoid public pressure or pressure of outsiders. The interview proceeded only if oral consent was given.

The survey took place in September and October 2016. It was conducted with the support of a local guide and interpreter who himself was a member of a rural-urban migrant family. Therefore, he had a deep cultural understanding and widespread network of contacts for potential interview partners. As an interpreter, he was proficient in English, Hindi, and Garhwali, the most common dialects spoken in the hill area. Initially, nine permanent migrant families were contacted to arrange interviews with the household head. The subsequent interview partners were recruited through a snowball system, which likely resulted in a skewed sample. In addition to the household visits, temple festivities, sports events and religious events such as Diwali (the Hindu celebration of lights to welcome Lord Ram) were attended to recruit further interview participants, and to gather insights in informal conversations. This deepened the cultural understanding, and thus also supported the interpretation of the survey results.

### Statistical analysis

The answers to the survey questions were digitalized into an Excel spreadsheet. The relations between two variables were analysed by a Chi^2^ test if the data were nominally scaled in both cases; if one variable was nominal and the other metrical, the Spearman correlation was calculated. To examine whether residuals of data were normally distributed, the Kolmogorov-Smirnov test was applied. The level of significance was set to p = 0.05 for all tests. When ordinal and metric variables were both present, a Spearman correlation test was carried out. Descriptive statistics were used to summarize the results.

### GIS-based land use analysis

The second part of the study comprised a Geographic Information System (GIS) analysis of land cover changes over the past two decades. The villages Kandai and Barkot, located in Pauri district, were chosen for a GIS-based time-series analysis because in these two villages respondents mentioned environmental change as a presumed general reason for migration. The selected villages were set as centre points of a 10 km x 10 km square, and the 100 km^2^ around them were analysed, based on a series of satellite images taken by Landsat 7 and 8 between 1980 and 2017 (provided by United States Geological Survey, Table 1). Land cover was classified using a supervised maximum likelihood classification, carried out with ArcMap. The initial classification distinguished eight different land use types: high-, medium- and low-density forest, pastures (with scattered trees or shrubs), fallow fields, standing crops, bare ground, and water bodies. An accuracy assessment was carried out for one exemplary year, 2011, following the procedure of Parece et al. [34]. The time-wise closest Google Earth image of 2012 was used for comparison. One hundred random points were spread over the selected area on both images and the classification results were verified with an accuracy of 70%. For mapping temporal and spatial changes in land cover maps, the eight initial classes were finally summarized to three major classes: ‘*other’* (no cover / water), *‘forest’* (high / medium / low density) and *‘agriculture’* (silvo-pastoral and agricultural fields).

**Table 1:** Acquisition dates of satellite images.

Satellite / sensor	Kandai	Barkot
Landsat 3		19.03.1980*
Landsat 5		16.03.1992
Landsat 5		11.03.1996
Landsat 5		20.03.1999
Landsat 7 ETM+	01.03.2001	01.03.2001
Landsat 7 ETM+	10.05.2003	10.03.2003
Landsat 5 TM	15.03.2009	15.03.2009
Landsat 5 TM	05.03.2011	
Landsat 8	13.05.2013	
Landsat 8	03.05.2015	17.04.2015
Landsat 8	02.03.2016	
Landsat 8	05.03.2017	05.03.2017

The selected images were analysed for land cover changes around the villages Kandai and Barkot in Uttarakhand, Northern India. Resolution is 30 m except for the oldest image of Barkot^(*)^ with 60m.

### Analysis of FSI forest reports

Changes in forest cover detected at the village scale were compared to the development reported by the Forest Survey of India (FSI) at district (Pauri, ca. 5,300 km^2^), state (Uttarakhand, ca. 53,500 km^2^), and national scale (India, ca. 3,290,000 km^2^). Data were extracted from the biennial “State of Forest Reports” [35] available on the website of the Ministry of Environment, Forest and Climate Change, Dehradun from 2001 onwards.

## Results

### Survey

#### Socio-economic profile of rural-urban migrants in Dehradun

Among the 100 migrant households interviewed in this study, rural-urban migration took place over an extended period of time, with the earliest record in 1955 and the latest in 2016. The migration flow was rather continuous and not marked by discrete peaks or discontinuities. The districts of origin were Tehri, Pauri, Uttarakhashi, Rudraprayag, Pithoragarh, Chamoli, Almora, and Dehradun. The total number of home villages covered in the survey was 69. They were unevenly distributed between the districts, with 33 villages in Tehri followed by Pauri with 15 villages of origin, and less than 10 in the remaining districts. The number of respondents from the same village was highest for Seeriyan with 13, and Goran with 8. Nine more villages were represented by 2 to 3 respondents, the rest by only a single household.

Household sizes ranged from one to eleven members, with an average of 4.6 household members. The gender balance of the household heads in this survey was 63 male and 37 female; 87 were married, 6 unmarried and 7 widowed. Of the 100 respondents 79 had migrated together with at least one other family member.

The system used for assessing the education within the migrant families distinguished five levels: 0—None; 1—primary school finished (5 years); 2—middle school finished (8 years); 3—secondary/high school/matriculation finished (10 years); 4—scholar or university education finished (more than 10 years). The overall educational background in the migrant households was high: 75% had reached level 4, 10% finished secondary/high school, 4% middle school, 7% primary school and another 4% did not receive any education at all. Five of the interviewees were illiterate and four of those were female. The average education level of the respondents’ families, including the household head and excluding children, was 3.5.

**Fig 2:**
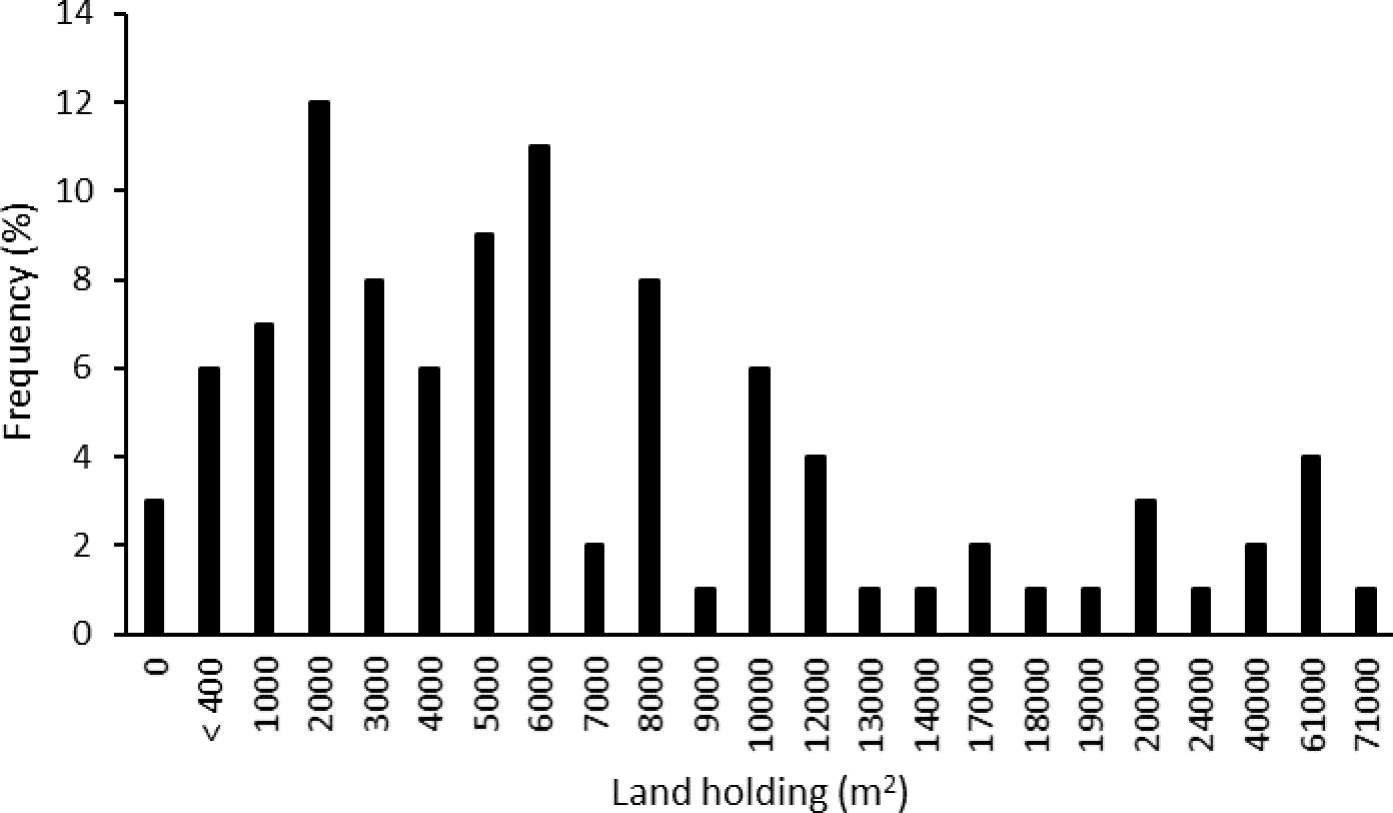
Size of land holdings of migrant families in their village of origin in Uttarkhand, Northern India. Most respondents (76%) owned property smaller than 1 ha.

In our survey, 97% of the migrants still owned land in their home villages, meaning that either themselves or their families did (Fig 2). The average land holding of all respondents was just below 1 ha (9662 m^2^); 21% had larger properties. Most of the respondents had been actively involved in agriculture before moving to the city; 85% had worked on their own fields, another 3% on their parents’ land.

#### Occupation before and after migration

**Fig 3:**
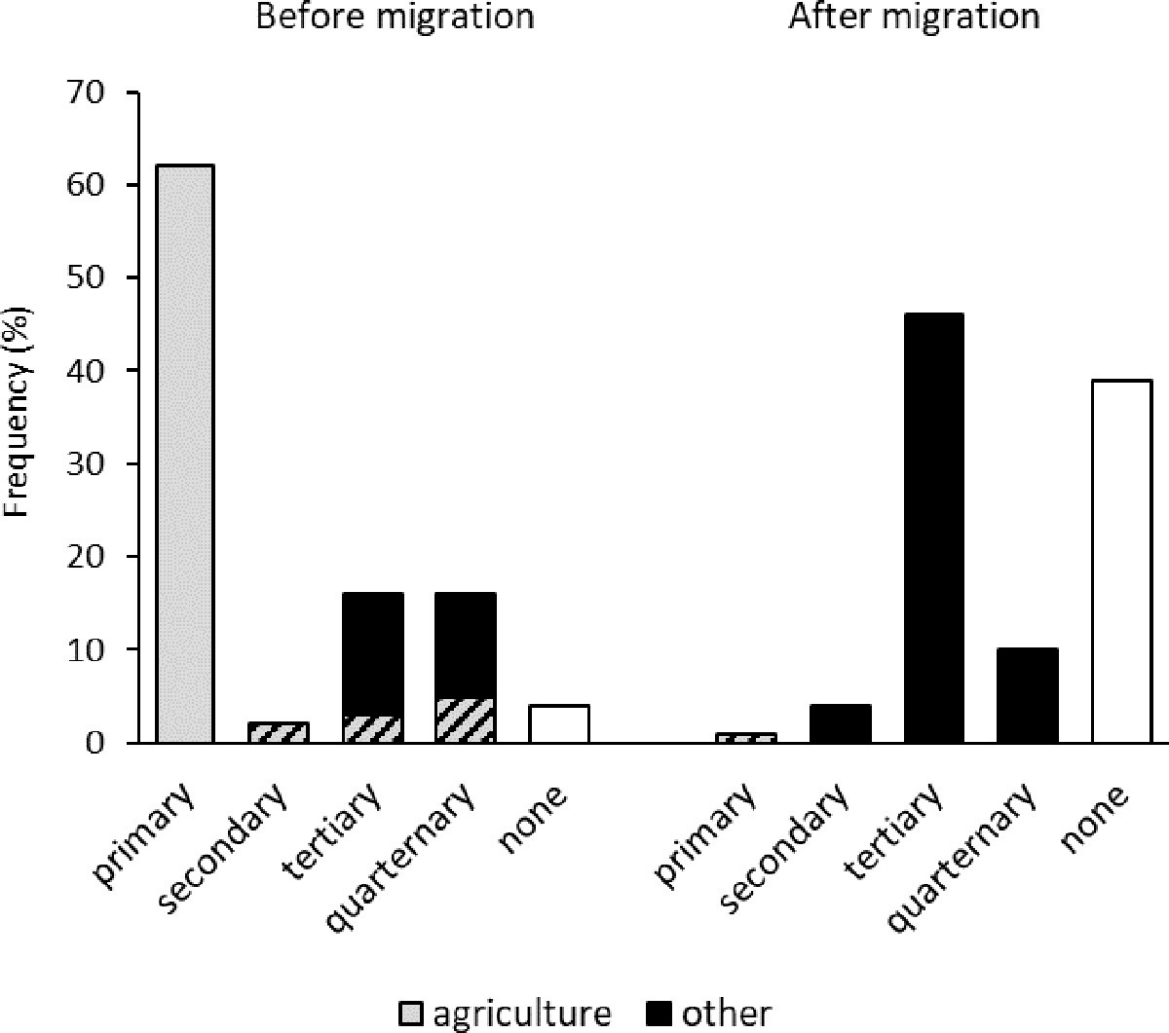
Occupational sector of the respondents before and after migration to Dehradun, Northern India.

The occupation pattern shows that rural-urban migration not only describes the geographical movement of a physical residence, but also a profound shift in lifestyle (Fig 3). Agriculture is a typical activity in the primary sector of the economy, and prior to their migration to Dehradun it was mentioned as the only occupation by 64 respondents, and in combination with other occupations by another 10. Silk production was the only reference to the secondary sector, and it was also mentioned in combination with agriculture. Occupations in the tertiary sector comprised government jobs (8), army (4), shops (2) and health care (1), whereas teachers (2) and students (13) were accounted for in the quarternary knowledge sector. Four women described themselves as house wives, which is not a formal economic sector.

After migration only one respondent was still occupied in agriculture, whereas the majority worked in the tertiary sector. Here, government jobs still predominated (15); the remaining activities, however, were highly diversified (nearly 20 different job denominations). The quarternary sector comprised teachers (5) and Hindu Priests (3), but, due to the time passed since migration and status as household heads, there were no students. The answers not allocated to a sector comprised house wives (23), retired household heads (15), and one unemployed person.

#### Household income before and after migration

The average household cash income in the village before migrating was 10,794 INR, the 5% trimmed mean was 8,974 INR. When considering the number of household members, the average income per person and day in the villages was 43 INR, the 5% trimmed mean was 36.4 INR. The national poverty line of rural areas in Uttarakhand was 29.3 INR per day in 2011/12 [36], and the share of people living below the national poverty line of Uttarakhand in rural areas was 11.6%. In this survey, 18% of the people had lived below the poverty line of 2011/12 before migration.

**Fig 4:**
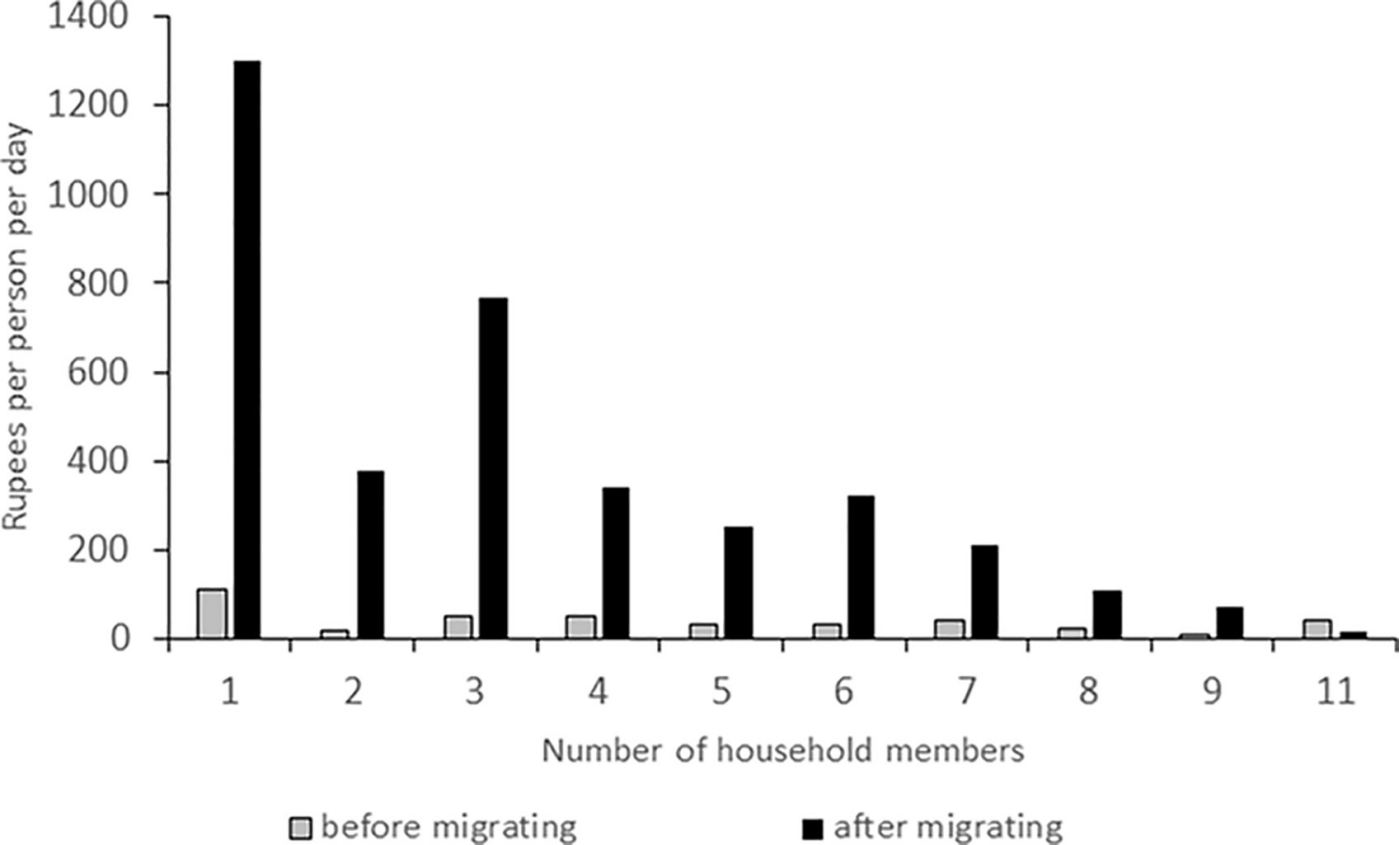
Income per person according to household size in Dehradun, Uttarkand, Northern India.

After migration the average mean income was 44,475 INR, and the 5% trimmed mean was 39,050 INR per household. This is almost four times as high as it was before migration and thus statistically highly significant (P = 0.001). This means that only 3% of the respondents now live in poverty, according to the urban poverty line of 36 INR per day per person for Uttarakhand in 2011/12. Thus, for poverty reduction, an improvement of 83.3% was achieved after migration. The average income per person per day rose to 398 INR, which represents a statistically significant difference of more than 10 times the income of that before migration (P<0.001). The 5% trimmed mean was 322 INR. The poverty line of 2011/12 serves here as an arbitrary reference for comparison although the respondents migrated in different years. It is obvious though (Fig 4), that the per person income improved from before to after migration for all household sizes except for the biggest household of eleven members. The results indicate a clear advantage for smaller households living in the city.

#### Personal reasons and perceived general reasons for migration

**Fig 5:**
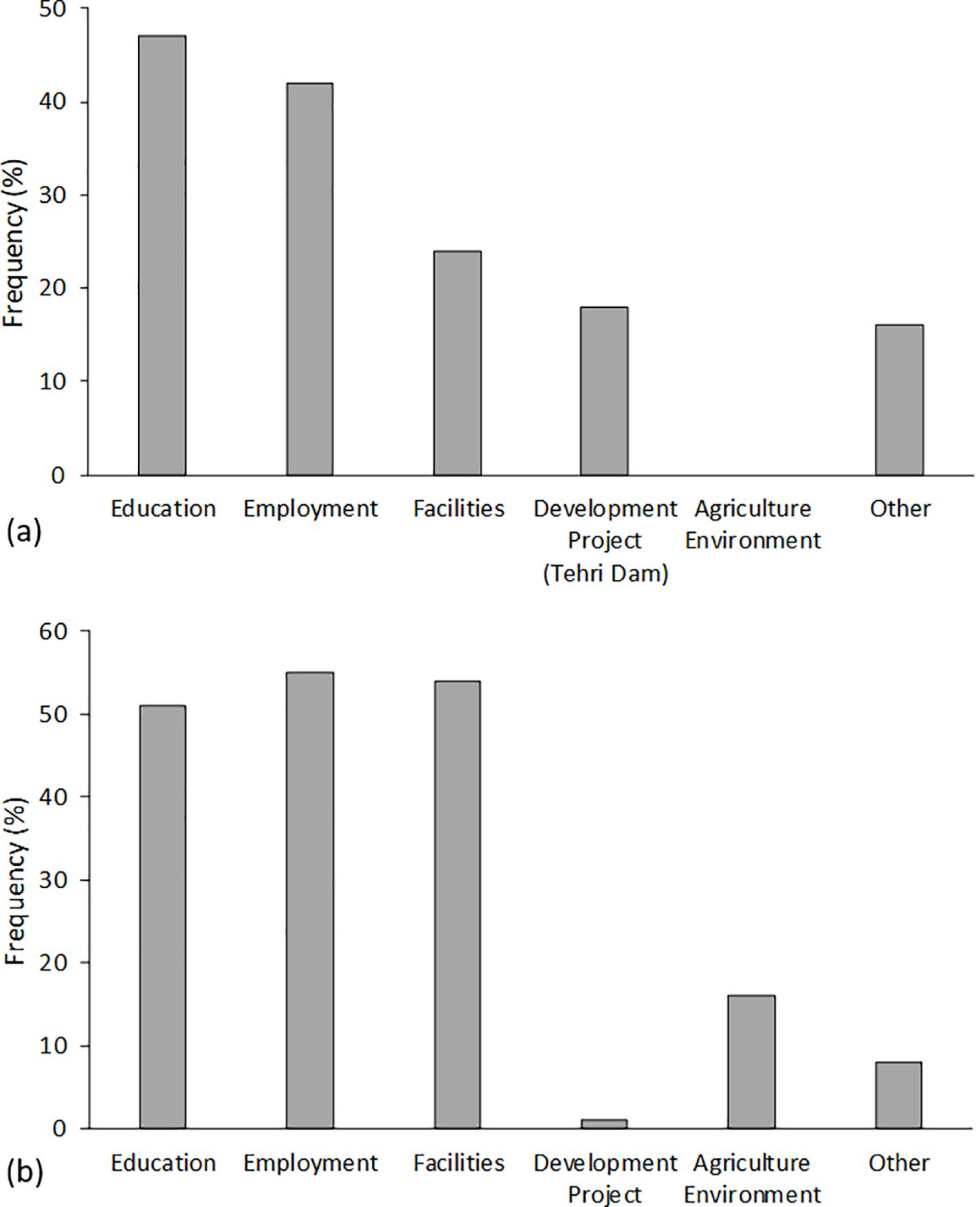
**Personal reasons (a) and perceived general reasons (b) for rural-urban migration in Uttarkhand, Northern India**.

The questions addressing the reasons for migration were open and several answers were possible. The main personal reasons mentioned by the respondents were, in order of relevance, education, employment (with its associated income), facilities, development projects (in particular the Tehri Dam) and others (Fig 5).

The educational institutions in the respondents’ villages of origin were usually limited to basic education. Even for elementary school, many villagers had to travel to another village. The poorly developed infrastructure made it difficult for many children to reach school. If a continuation of schooling was the goal, moving closer to the valley was often the only option. In our study 48 respondents mentioned their children’s or grand children’s education as one of their personal reasons to migrate. Women in particular seemed to have other household member’s educational future in mind when deciding to migrate.

The lack of employment opportunities in and around the villages was always mentioned in the context of the income to be achieved. Agriculture was seen as a risky activity with low profitability, which was unattractive to educated youth. Altogether, 42 respondents mentioned financial aspects related to employment as one of their reasons to migrate.

When facilities were mentioned, transportation infrastructure and health care such as hospitals and the presence of doctors were considered most important. In some cases, people moved away from their home because of health issues that could not be treated in their villages.

The construction of the Tehri Dam was mentioned as a personal reason for migration by 16 respondents, all of whom came from the drowned villages. Two more respondents referred to other projects such as road building. The category ‘other’ as a reason for migration includes life quality (7 occurrences), marriage, ties to already successfully migrated family members and other singular statements. It is noteworthy that in the open question none of the respondents mentioned environmental conditions as a reason to migrate.

When it came to the general reasons for migration in Uttarakhand, compared to the respondent’s personal reasons for migrating, the answers were similar (Fig 5). Employment was mentioned most frequently, then facilities (especially medical and transportation) and education. Only one respondent considered the Tehri Dam as a general reason for migration. In this context, however, some environmental factors were mentioned among the other reasons; these were natural disasters (5 occurrences), conflicts with wildlife (1), and declining soil fertility (11), summarized in Fig 5 in the column ‘agriculture / environment’.

It became apparent that the other respondents were also aware of environmental factors as well when they were prompted with a list of potential problems perceived in the village. The answer ‘problems with agriculture’ was confirmed by 52 respondents, 54 also confirmed landscape changes. In their further explanations the respondents mentioned landslides (27 times), drought (24), low productivity or low soil fertility (25), and conflicts with animals/wildlife (33) which either destroyed the crops (such as free-ranging cattle, wild pigs, or monkeys) or scared the farmers and kept them from attending to their fields (such as bears or tigers). The frequency of affirming the answers related to environmental factors was thus comparable to the confirmation of answers related to the main reasons for migration given in the open question, i.e. lack of educational opportunities (66), and financial issues (44) as major village problems. The top village problem was facilities, confirmed by 80 respondents. The predominant problem in the city was financial issues with 21 confirmations, whereas facilities and education problems were reduced to negligible levels, with 9 and 4 nominations, respectively.

#### Factors influencing reasons for migration

The factors presented in the socio-economic profile of the migrants were examined for their influence on the motivation for migration (Table 2). Due to the relatively small number of answers, the correlations’ significance was tested separately for six socio-economic variables against seven stated reasons for migration.

**Table 2:** Statistical analysis of dependencies between household characteristics and stated personal reasons for migration in Uttarkhand, Northern India.

	Education	Employment and income	Facilities	Development project (Tehri dam)	Life quality	Other
District of origin	P = 0.285	**P = 0.025**	P = 0,59 ^(^^2^^)^	**P = 0.007** ^(^^4^^)^	P = 0.346 ^(^^4^^)^	P = 0.352 ^(^^4^^)^
Household size^(^^1^^)^	P = 0.199	P = 0.695	P = 0.769	P = 0.154	P = 0.419	P = 0.334
Gender HHH	P = 0.8	P = 0.518	P = 0.67	P = 0.152	P = 0.197 ^(^^4^^)^	P = 0.628 ^(^^3^^)^
Educational level^(^^1^^)^ HHH	P = 0.393	P = 0.241	P = 0.526	P = 0.507	P = 0.784	P = 0.747
Land holding ^(^^1^^)^	P = 0.608	P = 0.068	P = 0.475	P = 0.156	**P = 0.017** cc = 0.240	P = 0.139
Involved in agriculture	P = 0.859 ^(^^4^^)^	P = 0.788 ^(^^4^^)^	P = 0.945 ^(^^3^^)^	P = 0.673 ^(^^3^^)^	P = 0.525 ^(^^3^^)^	P = 0.099 ^(^^3^^)^

^(1)^ Spearman correlation, all others: Chi^2^ test; cc = Correlation coefficient; significant influence is highlighted in bold.

^(2)^ 16.7% of the cells have an expected P value of <5

^(3)^ 25% of the cells have an expected P of <5

^(4)^ 33.3% or more of the cells have an expected P of <5.

The variables household size, gender of the household head, educational level, and time of migration (not shown) were not significantly related to any of the stated reasons for migration.

The amount of land owned in the village, however, was significantly correlated to the frequency of mentioning life quality (a component of ‘other’ in Fig 5A) as a reason for migration. The bigger the property, the more ‘life quality’ was mentioned as a reason for migration (P = 0.017). Mentioning employment as a reason was also more likely when more land was owned (P = 0.068), and it reached significance at an interval of 0.1. This suggested that, the more the respondents relied on agriculture in the home village, and the better their assets, the less they were satisfied with the life quality and employment/income generated from it. Due to the small sample size, however, the conditions for a Chi^2^ test were unfulfilled, and statistically this assumption could not be further supported.

For analysing the correlation between the respondent’s district of origin and the stated personal reasons for migration by a Chi^2^ test, the districts Chamoli, Almora, Rudraprayag, Pithoragarh, Dehradun and Uttarkashi, were combined into one category called ‘less represented districts’, while Tehri and Pauri represented one category each. Significant correlations were shown with the reasons employment (P = 0.025) and development project/Tehri Dam (P = 0.001). People coming from Tehri were more likely to mention development projects (P = 0.007) as a reason, whereas they were less likely to mention employment (P = 0.025). Respondents originating from Pauri, in comparison, were more likely to state employment as a reason (P = 0.025).

Finally, it was tested if the district of origin also influenced the reasons that were perceived as general reasons for rural-urban migration. However, the Chi^2^ test did not yield significant evidence (P = 0.231 to 0.695; data not shown). Other dependencies could not be tested because the conditions for the Chi^2^ test were not fulfilled.

#### Push *versus* pull forces

**Fig 6:**
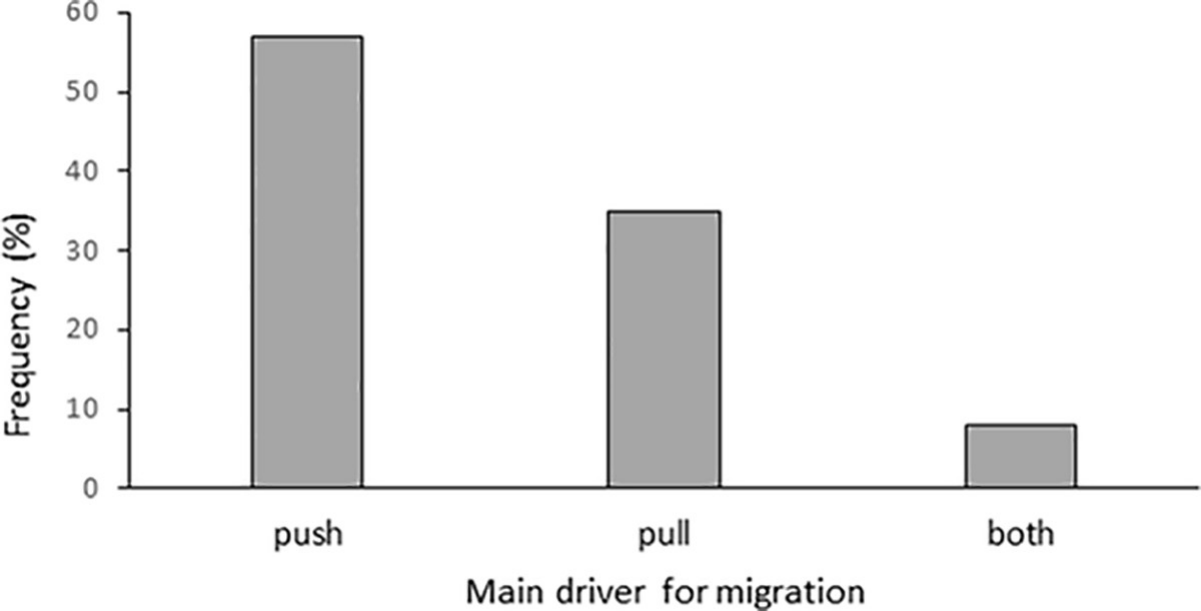
Push or pull factors driving rural-urban migration in Uttarakhand, Northern India.

After narrowing down the major drivers of migration, our respondents were asked if in their opinion it was a push or pull force that motivated the rural-urban migration (Fig 6).

The predominant feeling of being pushed out instead of pulled away indicates a rather negative association with the migration process and would support the common views of push-dominated migration. On the other hand, 85% of the respondents reported a positive change of their financial situation after migration, 10% did not see any difference and only 5% noticed worsening of their financial status (Fig 4). All five people perceiving a negative change of their financial situation believed that it was push factors that determined their migration decision. Out of the ten people feeling no difference in the financial aspect, 60% considered push factors more important, 30% pull factors, and the remaining person saw both factors as equally important. The district of origin did not significantly affect the opinion of whether it was push or pull factors that drove people to migrate from rural to urban locations within Uttarakhand (P = 0.843).

**Fig 7:**
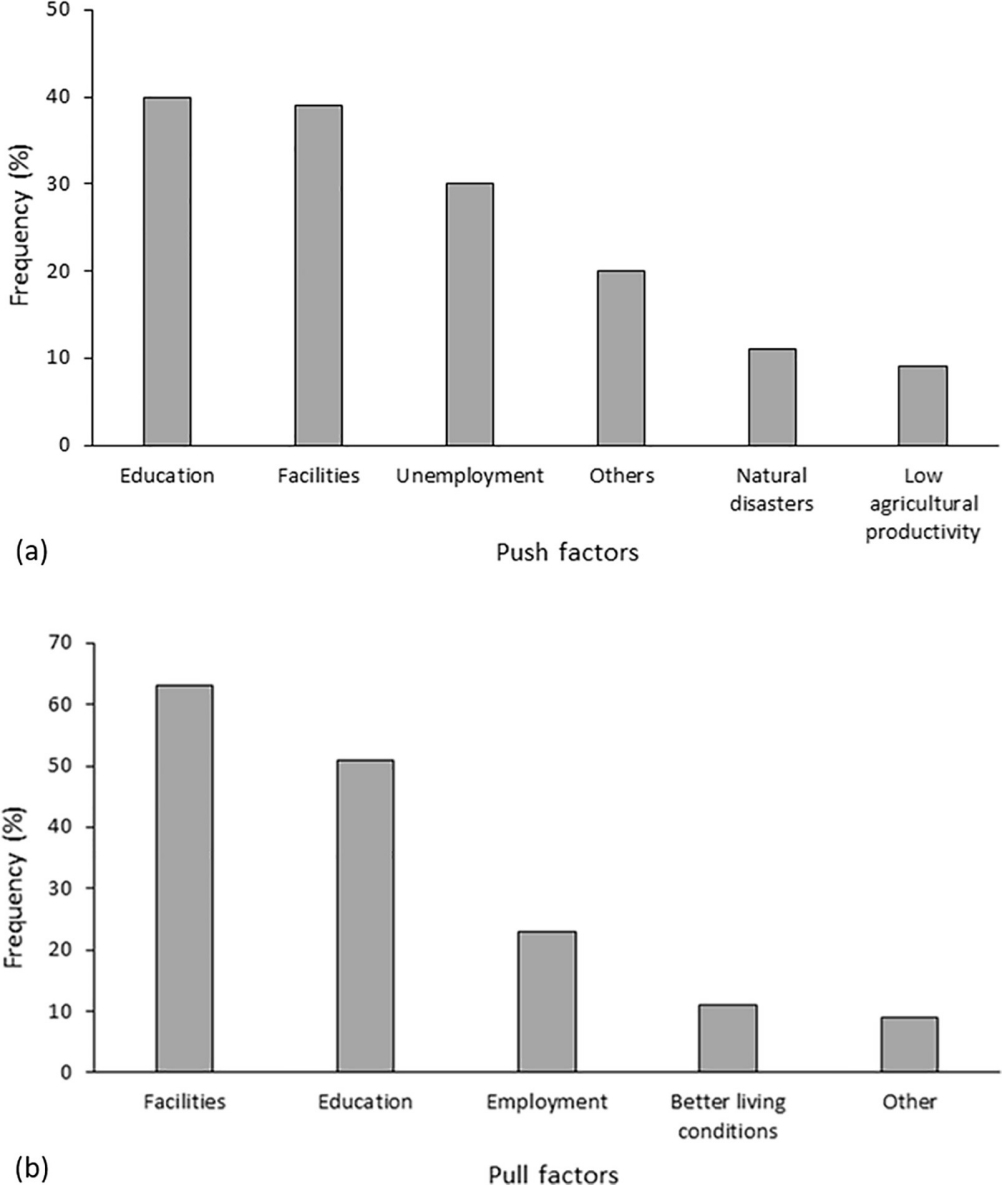
**Major push factors (a) and major pull factors (b) for rural-urban migration in Uttarakhand, Northern India**.

To further differentiate this result, the respondents were asked what the possible major push and the major pull factors driving people to migrate might be (Fig 7). Facilities, education and employment/income were similarly important as push and pull factors. Environmental conditions were low-ranking, and were mentioned primarily related to specific events, such as natural disasters or conflicts with wildlife, or indirectly as low agricultural productivity, which is well in line with the stated reasons for migration presented above.

### Analysis of land cover changes

#### Agricultural area and forest cover at the village scale

**Fig 8:**
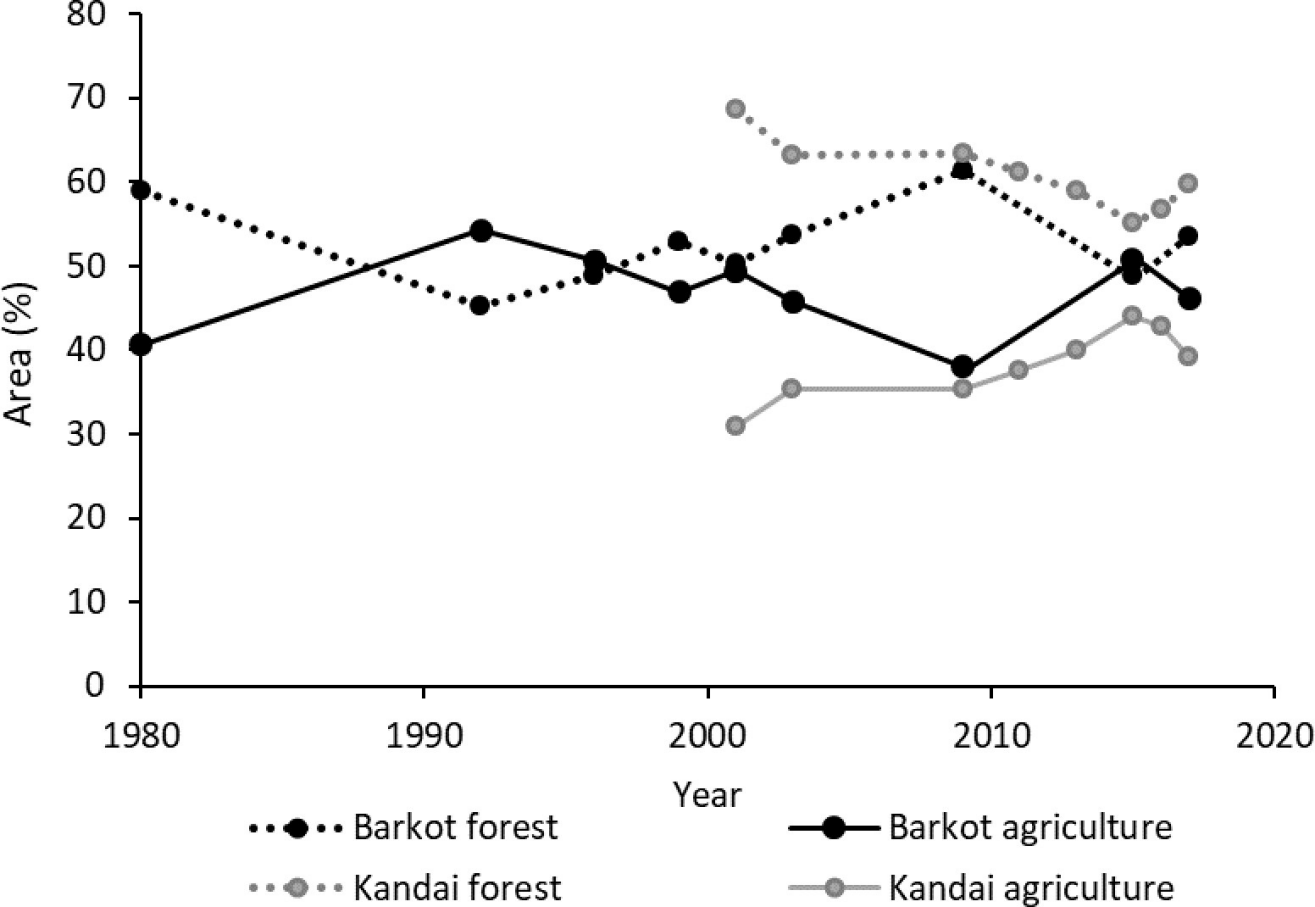
Agriculture and forest matrix of Barkot and Kandai villages, Northern India, over time.

None of the migrants stated an ecological reason as a personal reason for migrating, but they remembered environmental aspects when general reasons for migration or problems in the village were discussed. The second part of this study therefore focused on estimating by land cover analysis the degree of environmental change that actually took place and may have contributed to a rural push-driven migration. The most important aspect in land cover change connected to both landslides and agriculture was forest cover. Two villages were selected for this analysis: Kandai had a population of 86 people living in 22 households [16] and Barkot was inhabited by 174 people belonging to 38 families. Their agricultural fields were scattered around the village within an area of 10 km x 10 km in a largely forested area. The fluctuations in land cover were traced from 1980 to 2017 (Fig 8).

**Fig 9:**
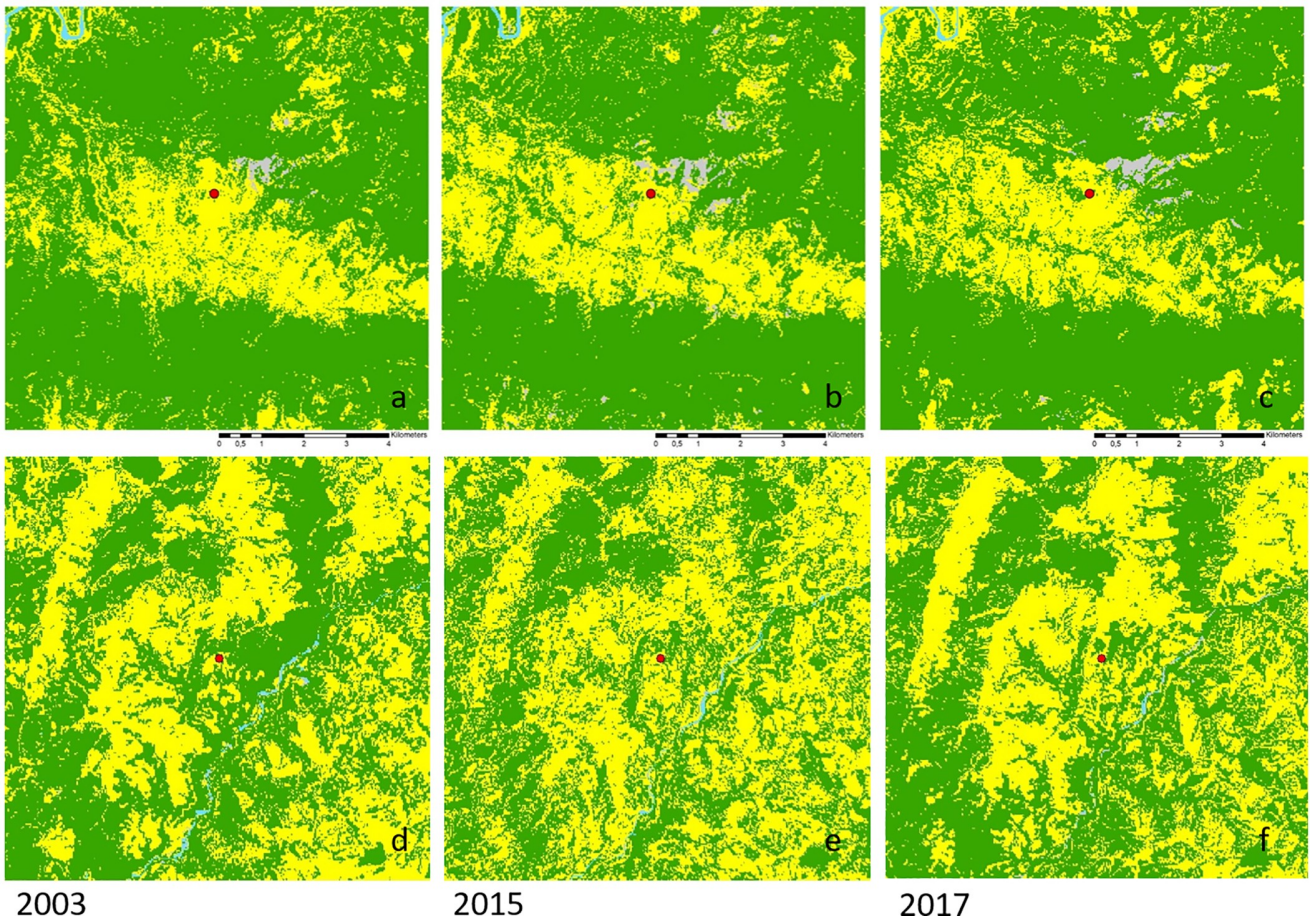
Land cover maps of the North Indian villages Kandai (a-c) and Barkot (d-f) in the years 2003, 2015, and 2017. Yellow–agriculture, green–forest, blue–water, grey–other; red dot–village center.

Since other land cover types represent only around 1% of the analysed area, the fluctuations in agricultural area just mirror those of total forest cover. The overall fluctuation was around 15%, with a minimum in forest area of 45% and a maximum of 61% in Barkot, and between 55% and 69%, respectively, in Kandai. The latter witnessed a steady loss of forest by 13.4% between 2001 and 2015, with this trend reversing only in the last two years by an increase of 4.5%. In Barkot, total forest area increased by 11% until 2009, but the entire gain was lost by 2015, and then recovered again to a value comparable to 2003. Since 2009, the two villages show similar trends. Even though the forest cover has not yet reached the level of previous peaks, it has increased since 2015 in both village surroundings. The land cover maps (Fig 9) show that from 2003 to 2015 agricultural areas in Kandai expanded mostly in the western part of the valley, and forest recovery between 2015 and 2017 was also most obvious in that area, as well as in an area north-east of the village. For Barkot forest cover changes were most evident in the river valley in the center of the image, close to the village.

**Fig 10:**
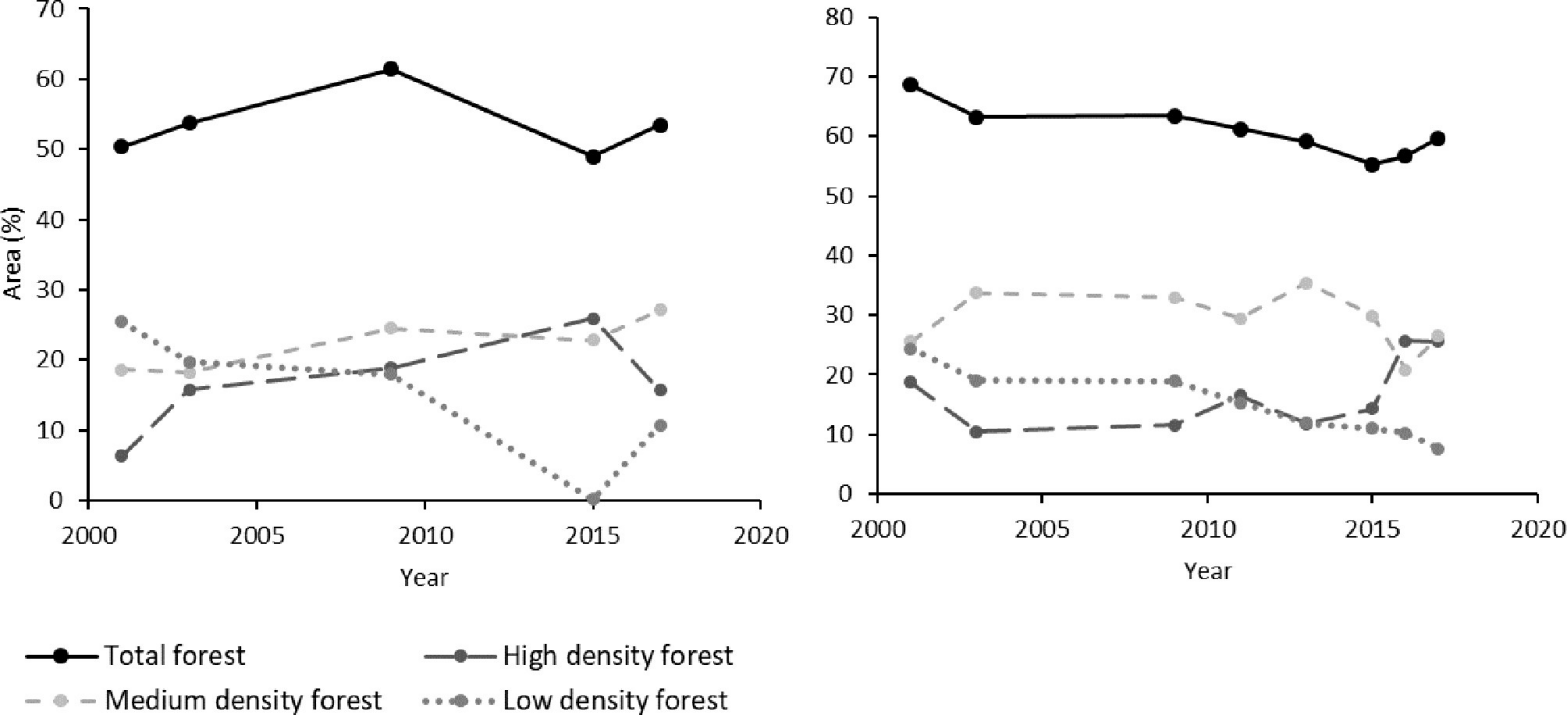
**Development of forest quality in a 100 km^2^ area around (a) Barkot and (b) Kandai, Uttarakand, Northern India, since 2000**.

When distinguishing high-, medium- and low-density forest, changes in forest quality can be estimated (Fig 10). In the areas around the villages, low-density forest tended to decline more than medium- or high-density forest. In Kandai the recent recovery was mostly due to an increase in high-density forest whereas in Barkot the increase in total forest was mostly due to low-density forest. The increase in high-density forest cover may indicate a more enduring and effective forest recovery.

#### Forest cover at larger scales

**Fig 11:**
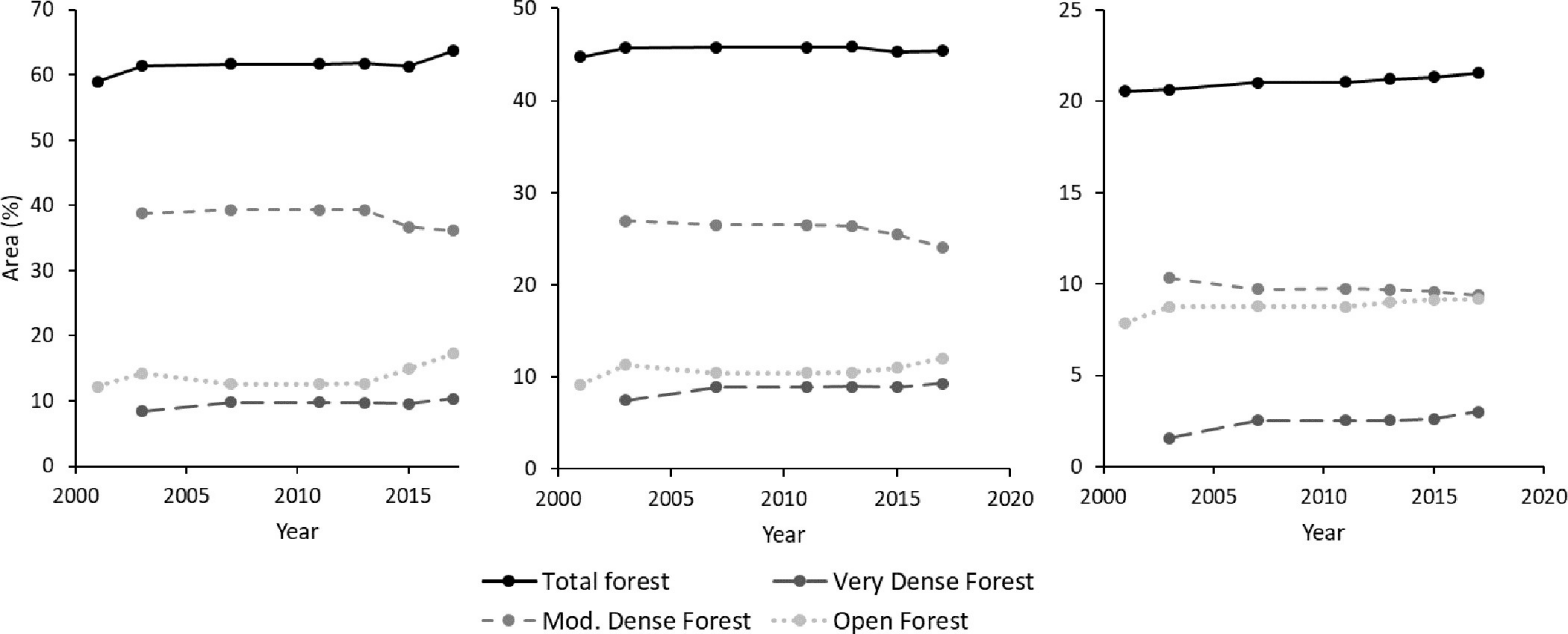
Development of forest cover and quality at different scales since 2000. (a) Pauri district (5,329 km^2^), (b) Uttarakhand state (53,483 km^2^), and (c) all of India (3,287,263 km^2^).

Since 2003, the forest survey of India differentiates between very dense, moderately dense, and open forest [35]. Though the results may not be directly comparable to the classification used above, unless they refer to total forest as the sum of all defined categories, the FSI data were analysed (Fig 11) to capture trends in forest cover changes that may not be detectable in the small-scale GIS-analysis.

From 2001 to 2017, the total forest cover in India, as well as in Uttarakhand, fluctuated marginally by 1%, with an overall increasing tendency. In the province of Pauri, a continuous increase of almost 5% was recorded. Some environmental deterioration was indicated by the quality of forest cover, and in all surveys the proportion of open forest increased at the expense of moderately dense forest. Very dense forest, on the other hand, was stable or increasing, though it had the overall lowest proportion, whereas moderately dense forest was most abundant. Thus, the narrative of severe deforestation being a significant rural push factor could not be substantiated for the time period analysed here, neither by the local analyses, nor by the FSI data.

## Discussion

The present study combined the methodologies of a socio-economic survey and a spatio-temporal land cover analysis to investigate the pattern and likely causes of rural-urban migration in Uttarakhand, a presumed example of rural push dynamics in Northern India. Although the sample of 100 households covered in the survey is rather small and, due to the snowball recruitment, certainly not representative, it contributes an original set of primary data to the scientific discourse. A unique feature of the current study is the approach of asking for personal (own) and general (other migrants’) reasons for migration, for problems perceived in the village and in the city, and for the respondents’ opinion on what is a push and what a pull factor. The ambiguities in the replies allow drawing some interesting conclusions.

### Push-pull factors and theories of migration

The predominant reasons for migration documented in this study were income/employment, education and facilities, which is well in line with previous studies [13, 15, 37]. These factors were mentioned by the respondents as personal and general reasons for migration, as major problems in the village, and as both, push and pull factors. Though the distinction of push and pull factors was generally understood by the respondents, it seemed to be arbitrary when linked to the personal migration decision. This finding warrants a closer look at how the underlying migration theories developed in the scientific discourse.

The early attempts to describe “laws of migration” date more than a century back and suggested mechanistic models based on aggregate population data, inspired by physical gravity laws [38, 39]. The more comprehensive concept of Lee [23] acknowledged that migration depends on positive and negative factors at the place of origin and destination, intervening obstacles, and personal factors. He presumed a limited flow of information, as he considered the positive and negative factors at the place of origin to be well known to the migrants, whereas their knowledge of the situation at the destination was incomplete. However, he pointed out, that the most overwhelming reasons for migration were usually of economic nature, and that net migration is highest if negative factors predominate at the place of origin, which in the socio-economic context of his time can be read as rural push-urban pull, although he did not formally introduce this terminology.

In the dual economy models [40, 41] positive and negative factors typically reflect the relative strength of the local economies, and rural-urban migration actually contributes to the transition from a (stagnating) economy based on a rural agricultural sector to a (growing) economy based on a diversified urban sector. Here it is primarily a difference in the supply and demand of labour, and in particular a high urban demand for labour, that drives migration until some kind of saturation is achieved. All of these push-pull theories of migration do not look at individual migration decisions, but instead rely on various aggregated socio-economic data and their uneven spatial distribution giving rise to (passive) flows of migrants. The mathematical modelling of spatial flows resulting from push-pull was presented by Dorigo and Tobler [42], whereby they explicitly note: “Instead of attempting to include complex human behavior in our equations, we estimate the effects of this behavior, allowing other researchers to estimate the relation of the computed pushes and pulls to life situations” (p. 14).

Subjective migration choices were first taken into account by behavioral models, such as the stress-threshold model [43], inspired by economic cost-benefit analyses, or behavioral-cognitive approaches [44] considering not only the economic aspect but also personal or household characteristics, individual goals, and societal norms. Both models were based on rational choice assumptions, but were subsequently criticized for being vague. Subjective migration choices can give rise to larger migrant flows by cumulative and circular causation [45] when information on successful migration is transmitted back to the place of origin, and past migration alters the context in which current migration decisions are taken. In this line of argument external factors are often neglected.

The present study has elements of both types of migration theories. In the questionnaire section it explores the individual migration decisions, while aiming to verify corresponding external factors in the land use analysis. The answers given by the respondents in the present study show very clearly the predominance of economic reasons for migration: employment/income and education (motivated also by aspiration for better paid jobs) were most frequently stated. This can be accounted for as a push factor if it is insufficient in the rural place of origin, and as a pull factor if it is available in the city. Facilities, the third major reason for migration, seemed to be prioritized as a component of a convenient urban lifestyle. Again, underdeveloped facilities in the rural areas would be as much a push factor as they are a pull factor to the city, where facilities are readily available.

In a field survey in 2013, which included 18 villages and a total of 217 households from Pauri, Garhwal and Almora districts, Mamgain and Reddy [13] reported that 88% of the sampled households had at least one member who emigrated for employment. Sati [15] presented a similar survey of 42 household heads in two villages within the district Chamoli in 2014. Both studies combined their results with secondary data from governmental surveys and conclude that rural-urban migration in Uttarakhand is entirely driven by push-factors. Here, however, it is often ambiguous from which part of the study these conclusions are drawn. Moreover, the questions were framed “in terms of the push factors and other constraints that impact out-migration” [15], so it is not surprising that the respondents (here the families residing in the villages) confirmed the researcher’s assumptions. In our study we observed that in the generic question a majority (58%) replied that push-forces were more important, but this was strongly related to their own degree of economic success. Those who were better off, tended to consider pull-factors more important. When disaggregating the factors, they were considered equally important as both, push and pull.

### Environmental push factors of migration

The fact that environmental push-factors had such a low priority in the present study was surprising. Apparently, they did not play any role in the personal migration decisions. That the respondents still mentioned them as a presumed general reason for migration may indicate a repercussion of established public narratives. When prompted with answers this became even more evident, as environmental problems received a high degree of confirmation, although the actual phenomenon of landscape change, and in particular deforestation, was hardly apparent in the land cover analyses.

The discourse on deforestation as a major rural push force goes back to the early 1990s when numerous accounts of growing environmental problems in developing countries were published, both in the popular press and in scientific research, such as the State of the World Reports 1988 to 1997 (e.g. 1989 [46], 1991 [47]). In response to this, Bilsborrow [8] analysed to what extent population growth and rural out-migration may affect land-use patterns and lead to deterioration of the natural resource base, in particular deforestation. His approach was a cross-country comparison, based on data from the World Resources Institute (WRI [48]), and focused on a 12 year period in the 1980s, a time frame set to capture contemporary links between the chosen parameters. Due to many confounding issues, however, only weak relationships between population dynamics, agricultural land use and deforestation were shown. Nevertheless, subsequent research continued building on his hypotheses, with contradictory results. Whereas Black [32] did not find sufficient evidence for the connection between landscape changes and rural-urban migration, de Weerdt and Hirvonen [49] found proof for environmental degradation as a push factor and stated reason for migration in Tanzania. The term “disaster migration” introduced by Krecek et al. [33] exemplifies the causal relationships often hypothesized between landscape changes and migration, which, however, could not be confirmed in the present study.

According to Bilsborrow [8], within Asia, India and Nepal had the highest rates of deforestation reported to WRI with over 4% loss per year during the 1980s. The Forest Survey of India [35], used for the forest cover analysis in the present study, started its records in 1987; it reported a decrease in forest area all over India of 1.16% over 10 years until 1997, and after that a recovery of 11.8% until 2017, likely the result of strictly enforced legislation and the introduction of natural gas for cooking to even remote areas. The state of Uttarakhand was only founded in 1997, and since then had a rather stable forest area. The apparent contradictions may thus be due to the different time scope analysed. Nevertheless, declining soil fertility, droughts, and on the other hand extreme weather events causing landslides and flash floods are well documented in the study region [13, 14] and seriously affect agriculture, worsening poverty and urging people to seek alternative income sources [13, 50].

Slow and gradual environmental changes, however, for example in forest integrity or climate, were perceived but not prioritized by the village farmers in the present survey. If at all, environmental push was reported in relation to rapid changes (development projects) or sudden incidents/events (landslide). Shortcomings in agriculture were often framed economically (insufficient income, low productivity), but drought and low soil fertility were also frequently mentioned as problems. A straightforward ecological framing (disturbed ecological balances/cycles, declining biodiversity) was never stated. On the contrary, encounters with wildlife were reported as a major problem, because wild animals destroy crops and threaten human lives. At the same time, it is forbidden to kill them in India’s protected areas. This may indicate that modern, ‘urban’ mindsets are already displacing traditional values even in the remote villages, and people seek urban lifestyles to satisfy their needs and well-being.

### Service choices and changes in lifestyle

According to the ecosystem services framework [51], a healthy natural environment can deliver a range of services to satisfy human needs and maintain human well-being. It will provide food, feed, and timber, regulate temperature and water cycles, prevent (to some degree) flooding or landslides, and provide cultural services in terms of aesthetics, recreation, or religious practices. In rural societies people directly access these services in their daily lives and often feel an emotionally tight link to nature. In the urban environment the migrants will increasingly rely on non-ecosystem services for their personal well-being [52]. This is also what the respondents in the present study actually seek, as indicated by the high rank of facilities as a reason for migrating, or by very generic answers such as ‘life quality’ or ‘attractiveness of the city’ as reasons for their migration decision. These findings are well in line with the conclusions of van der Land [53]. Failing ecosystem services due to environmental degradation are much less important to the rural-urban migrants, than the active aspiration for a modern, urban lifestyle. In contrast to earlier times, migrants are well-informed about the conditions in the cities by the global media and telecommunication systems.

The present study’s lack of statistically significant interactions between socio-economic characteristics of the households and the personal reasons for migration points in the same direction, i.e., decisions motivated by hopes and aspirations rather than certain vicious cycles. The interaction between the reason ‘Development project’ and the provenance of the respondents was due to the large cluster of migrants displaced by the Tehri dam. The interaction between the size of land holding in the village and the reason ‘Life quality’ relies on a fairly small number of answers. Nevertheless it may indicate that the better off the respondents in the village already were, the more they strived for yet further improving their socio-economic situation towards modern urbanity.

Migration not only has a spatial (“horizontal”) dimension of moving from one place to another, but also entails a socio-economic (“vertical”) dimension [54] in terms of a change in lifestyle. In the current survey, this was evident in the occupation profile before and after migration, which changed from an agriculture-dominated one to a highly diversified profile with a majority of people working in the tertiary service sector. This is indicative of a green loop and a red loop society, respectively [52], and thus exemplifies a rural-urban transition in the individual biographies.

### Outcome of migration for the migrants

Migration was successful for most of the surveyed households which is consistent with most other studies [13, 21, 37]. A recent survey of urban in-migrants in Dehradun and Haridwar [55], which reported that they were poorly educated, often unemployed and prone to urban poverty, is not comparable to our study, as it focused on slum-dwellers and comprised mostly inter-state, seasonal migrants.

Whereas, in the present study, employment and education were mentioned as major problems in the village, their share in ‘problems in the city’ was negligible. For most respondents the individual and household income had increased dramatically after migration. Only one of the respondents was unemployed; on the other hand, after migration 38 persons stated that they were either retired or house wives, whereas this answer was rarely given for the situation before migration, which is most likely because all family members still contributed to agriculture, even when getting married or of age. The overall convenience of life in the city had been described above. Nevertheless, despite these positive outcomes, financial issues were still the major problem in the city, and when asked about their emotional ties to the village of origin, many of the respondents said they would like to migrate back to their rural area of origin if they could. Among the group originating from the villages affected by the Tehri dam project, this answer was even more frequent.

Tehri constitutes a special case, as a large hydropower project was implemented in this province, with the construction of the Tehri dam between 1994 and 2006. It resulted in an artificial lake of 52 km^2^ surface area which in 2004 drowned or seriously affected several villages (Seeriyan, Malideval Chat Saur, Ghansali and Tipri). To compensate the inhabitants, the model town of New Tehri was established by the government, along with an associated employment scheme at the new location. In this case people were thus forced to migrate, and were only free in choosing Dehradun (rather than New Tehri) as their destination.

### Outcome of migration for the rural environment

The limited sample of 100 migrants surveyed in this study does not allow far-fetched conclusions regarding the effects of migration on the environment in the area of origin. It was difficult to even choose exemplary villages for the land cover analysis, since neither a temporal peak of migration was noted, nor a spatial clustering apart from the villages affected by the Tehri dam. The justification for choosing Kandai and Barkot for the land cover analysis was thus rather weak, but the composition of the sample was not known *a priori*, due to the snowball recruitment. Many villages were represented by only a single household interviewed in Dehradun, and nothing is known about the overall out-migration from these villages. Nevertheless, in the two exemplary villages Kandai and Barkot, the fluctuations in land cover pointed towards a period of expanding agricultural areas at the expense of forest until 2015, followed by reversal of this trend to date. With due precautions, this may be seen as a small but positive impact of out-migration on the environment, in terms of forest recovery.

## Conclusions

The hypothesis of the present study, that ‘*People of rural Himalayan Uttarakhand are pushed to migrate to the city due to environmental change*’ was not supported by the data presented above; neither were environmental reasons relevant for the migration decisions, nor could extensive deforestation be shown in the land cover analyses at different scales. Likewise, the push-pull paradigm proved inadequate to explain the migration decisions of the respondents interviewed, as the perception of a factor as push or pull depends on the perspective. There is clearly a large gap between rural and urban locations in certain parameters such as education opportunities, employment, income, and facilities, which may be better conceptualised as a gradient. These parameters could even be described by quantifiable indices. The driving force for migration then results from the width of this gap, or steepness of the gradient between the rural and urban settings. People decide to migrate if a threshold is passed in a priority factor, or in combinations of several of them. The thresholds, however, and actually even the direction of migration, are not determined externally, but depend on the individual perception of well-being, and thus ultimately on the personal and societal values. Such a gradient-threshold concept might allow linking the different strands of migration research, by employing aggregate statistics for describing the gradient, and sociology, behaviour and decision-making for setting the threshold. Scenarios of external conditions under which people migrate would thus be systematically related to the migrants’ attitudes and values.

## Supporting information

S1 File Survey questionnaire. (PDF)

S2 File Participant information for obtaining oral consent. (PDF)

## References

[1] Seto KC, Parnell S, Elmqvist T. A global outlook on urbanisation In: Elmqvist T. et al (eds.) 2013 Urbanisation, Biodiversity and Ecosystem Services: Challenges and Opportunities. A Global Assessment 2013. 10.1007/978-94-007-7088-1_1.

[2] Elmqvist T, Redman CL, Barthel S, Costanza R. History of urbanisation and the missing ecology Chapter 2, In: Elmqvist T. et al (eds.) 2013 Urbanisation, Biodiversity and Ecosystem Services: Challenges and Opportunities. A Global Assessment, 13–30. 2013. 10.1007/978-94-007-7088-1_1

[3] Angel S, Sheppard SC, Civco DL. The dynamics of global urban expansion. Washington (DC): Transport and Urban Development Department, The World Bank; 2005.

[4] Glaeser E., Henderson J.V. 2017 Urban economics for the developing world: An introduction. Journal of Urban Economics 98, 1–5.

[5] Bren d’Amour C, Reitsma F, Baiocchi G, Barthel S, Güneralp B, Erb K-H, Haberl H, Creutziga F, Seto KC. Future urban land expansion and implications for global croplands. PNAS. 2017; 114: 8939–8944. 10.1073/pnas.1606036114 28028219 PMC5576776

[6] Parés-Ramos IK, Gould WA, Aide TM. Agricultural abandonment, suburban growth, and forest expansion in Puerto Rico between 1991 and 2000. Ecology and Society. 2008; 13: 1 [online] URL: http://www.ecologyandsociety.org/vol13/iss2/art

[7] Singh PV. Changing trends of Agricultural development and its effect on environment of Uttarakhand Hills (A challenge). Scholarly Research Journal for Interdisciplinary Studies. 2017; 4: 8608–8622. 10.21922/Srjis.V4i37.10824

[8] Bilsborrow RE. Population growth, internal migration, and environmental degradation in rural areas of developing countries. European Journal of Population. 1992; 8: 125–148. 12158965 10.1007/BF01797549

[9] Bilsborrow RE. Migration, population change, and the rural environment. ECSP Report. 2002; 8: 69–94. http://wilsoncenter.org/sites/default/files/Report_8_BIlsborrow_article.pdf.

[10] Rais M, Pazderka B, Vanloon G. Agriculture in Uttarakhand, India—Biodiversity, nutrition, and livelihoods. Journal of Sustainable Agriculture. 2009; 33: 319–335.

[11] Guha R. The unquiet woods Ecological change and peasant resistance in the Himalaya. Expanded Edition Bangalore: University of California Press; 2000.

[12] India LEAD. Valuation of ecosystem services and forest governance A scoping study from Uttarakhand. New Delhi: LEAD India; 2007.

[13] Mamgain RP, Reddy DN. Outmigration from hill region of Uttarakhand: Magnitude, Challenges and Policy Options Final Report. 2015 National Institute of Rural Development (NIRD), Hyderabad.

[14] Satendra AK, Gupta VK, Naik TK, Roy S, Sharma AK, Dwivedi M. Uttarakhand Disaster 2013. New Delhi: National Institute of Disaster Management, 2015.

[15] Sati VP. Patterns and implications of rural-urban migration in the Uttarakhand Himalaya, India. Annals of Natural Sciences. 2016; 2: 26–37.

[16] Government of India, Ministry of Home Affairs [Internet]. Census of India 2011. [cited 2018 Mar 15]. Available from: http://censusindia.gov.in/2011-Common/CensusData2011.html.

[17] Haigh M, Rawat JS. 2012 Landslide disasters: seeking causes: a case study from Uttarakhand, India pp 218–253. In Krecek J, Haigh M, Hofer T, Kubin E. (eds) Management of Mountain Watersheds. Dordrecht, NL: Springer, and New Delhi, India: Capital (Co-publishers). ISBN 978-94-007-2475-4.

[18] Sridhar KS. 2016. Costs and Benefits of Urbanization: The Indian Case. ADBI Working Paper 607. Tokyo: Asian Development Bank Institute. Available: https://www.adb.org/publications/costs-and-benefits-urbanization-indian-case

[19] Denis E, Zérah M-H. 2014. Rural-Urban Linkages: India Case Study. Research Report 124. Rimisp, Santiago, Chile: Centro Latinoamericano para el Desarrollo Rural, 2014. halshs-01104630

[20] Chandrasekhar S, Sharma AJ. Urbanization and Spatial Patterns of Internal Migration in India WP-2014-016. Mumbai, India: Indira Gandhi Institute of Development Research, 2014.

[21] Hnatkovska V, Lahiri A. Rural-urban migrants in India: 1983–2008. The World Bank Economic Review. 2015; 29 10.1093/wber/lhv025

[22] Dutta D, Kundu A, Rahman A. Growth of Dehradun city: An application of linear spectral unmixing (LSU) technique using multi-temporal landsat satellite data sets. Remote Sensing Applications: Society and Environment. 2015; 1: 98–111.

[23] Lee ES. A theory of migration. Demography. 1966; 3: 47–57.

[24] Harris JR, Todaro MP. Migration, unemployment and development: A two-sector analysis. The American Economic Review. 1970; 60: 126–142.

[25] The World Bank. Global Monitoring Report 2013: Rural-Urban Dynamics and the Millennium Development Goals Chapter 2: Rural-urban disparities and dynamics. Pp 85–126. Washington, DC, USA: The World Bank, 2013. ISBN: 978-0-8213-9806-7.

[26] Jedwab R, Christiaensen L, Gindelsky M. Demography, urbanization and development: Rural push, urban pull and … urban push? Working Paper. Washington DC, USA: Department of Economics, George Washington University, 2014. http://home.gwu.edu/~jedwab/JGC_Sept2014.pdf

[27] Sridhar KS, Reddy AV, Srinath P. 2010. Is it push or pull? Recent evidence from migration in India. Final Report No. 10–04. South Asia Network of Economic Research Institutes, 2010. http://saneinetwork.net/Files/10_04___K_S_Sridhar.pdf.

[28] Rhoda R. Rural development and urban migration: Can we keep them down on the farm? International Migration Review. 1983; 17: 34–64. 12339122

[29] Tacoli C. Rural-urban interactions: A guide to the literature. Environment and Urbanization. 1998; 10: 147–166.

[30] Vinayakam K, Sekar SP. Rural to urban migration in an Indian metropolis: Case study Chennai city. IOSR Journal of Humanities and Social Science. 2013; 6: 32–35.

[31] Hagen-Zanker J. Why do people migrate? A review of the theoretical literature. Maastricht Graduate School of Governance, Working Paper No. 2008/WP002; 2008.

[32] Black R. Refugees, environment and development. New York: Addison Wesley Longman Limited; 1998.

[33] Krecek J, Haigh MJ, Hofer T, Kubin E. Management of Mountain Watersheds. New Delhi: Capital Publishing Company; 2012.

[34] Parece T, Campbell J, McGee J. Remote Sensing in an ArcMap Environment.2^nd^ edition Virginia View; 2017 Online version available from: http://www.virginiaview.net/education

[35] Government of India, Ministry of Environment, Forest and Climate Change [Internet]. Forest survey of India. [cited 2018 Apr 25] Available from: http://fsi.nic.in/details.php?pgID=sb_64.

[36] Government of India, Ministry of Electronics and Information Technology, National Informatics Centre. Open Government Data Platform, India [Internet]. Population below poverty line. [cited 2018 Feb 25]. Available from: https://data.gov.in/catalog/below-poverty-line-india.

[37] Jain A. Labour migration and remittances in Uttarakhand: case study report. Kathmandu: International Centre for Integrated Mountain Development, 2010.

[38] Stewart JQ. Demographic gravitation: evidence and applications. Sociometry 1948; 11: 31–58.

[39] Ravenstein EG. The laws of migration. Journal of the Statistical Society of London. 1885; 48: 167–235.

[40] Lewis WA. Economic development with unlimited supply of labour. The Manchester School. 1954; 22: 139–191.

[41] Piore MJ. Birds of passage: migrant labor and industrial societies. New York: Cambridge University Press, 1979.

[42] Dorigo G, Tobler W. Push-pull migration laws. Annals of the Association of American Geographers. 1983; 73: 1–17.

[43] Wolpert J. Behavioural aspects of the decision to migrate. Papers of the Regional Science Association. 1965; 15: 159–169.

[44] Crawford T. Beliefs about birth control: a consistency theory analysis. Representative Research in Social Psychology. 1973; 4: 53–65. 12306857

[45] Massey DS. Social structure, household strategies, and the cumulative causation of migration. Population Index. 1990; 56: 3–26. 12316385

[46] Worldwatch Institute. State of the world report 1989. Washington, DC, USA: Worldwatch Institute, 1989. ISBN: 0-393-30567-8

[47] Worldwatch Institute. State of the world report 1991. Washington, DC, USA: Worldwatch Institute, 1991. ISBN: 0-393-30733-6

[48] World Resources Institute, 10 G Street NE Suite 800, Washington, DC 20002, USA. Founded 1982. https://www.wri.org/

[49] de Weerdt J, Hirvonen K. Risk sharing and internal migration. Policy Research Working Paper No. 6429. Washington (DC): World Bank; 2013.

[50] Aggarwal J, Agrawal S. Uttarakhand. Past, present and future. New Delhi: Concept Publishing Company; 1995.

[51] Millennium Ecosystem Assessment Ecosystems and human well-being: synthesis. Washington (DC): World Resources Institute. Island Press; 2005.

[52] Cumming GS, Buerkert A, Hoffmann EM, Schlecht E, von Cramon-Taubadel S, Tscharntke T. Implications of agricultural transitions and urbanization for ecosystem services. Nature 2014; 515: 50–57. 10.1038/nature13945 25373674

[53] van der Land V. The environment-migration nexus reconsidered: why capabilities and aspirations matter [dissertation]. Frankfurt: J.W. Goethe-Universität, am Main; 2015.

[54] Mabogunje AL. Systems approach to a theory of rural-urban migration. Geographical Analysis 1970; 2: 1–18.

[55] Kandpal SD, Kakkar R, Aggarwal P, Roy D. Socio-demographic determinants in urban ‘in-migrants’ of Uttarakhand. National Journal of Community Medicine. 2018; 9: 463–468.

