# Supplementary material for: Is the push-pull paradigm useful to explain rural-urban migration? A case study in Uttarakhand, India
Source: PLoS One. 2019 Apr 2;14(4):e0214511. doi: 10.1371/journal.pone.0214511 (PMC6445429; doi:10.1371/journal.pone.0214511)
Supplement: S1 File — (PDF) [file pone.0214511.s001.pdf]

ANNEX

A1. Questionnaire

**Questionnaire number**			
**Starting / ending time**			**Duration:**
**Neighbourhood**			

RURAL-URBAN MIGRATION

This questionnaire includes several questions about your migration from a rural area to Dehradun. It is part of my Master´s thesis and I will highly appreciate your participation in my research. By answering these questions, you will help me a lot to carry out my work. Thank you very much!

Household head (HHH)

Name Age

Sex

☐ 1 Male ☐ 2 Female

Origin (rural area)

Year of migrating from rural to urban area

Marital Status

☐ 1 Married ☐ 2 Single ☐ 3 Widowed ☐ 4 Divorced

Occupation before migrating

Occupation now

Literate?

☐ Yes | ☐ No

Education

☐ 0 None ☐ 1 Primary (5 years) ☐ 2 Middle (8 years)

☐ 3 Secondary/ High School/ Matriculation (10 years) ☐ 4 More than 10 years

Household (HH) members (including everybody, also HH head)

By age	0-15		16-55		> 55		Total no.
Sex	m	f	m	f	m	f	
Number							
Literate?	yes	no	yes	no	yes	no	
Education level (0-4)							

Reasons for and consequences of migrating

Did you come alone?

☐ Yes No, I came with ☐ My husband / wife ☐ My children ☐ Other____________

Why did you decide to move?

Did/ do you own land in the area you migrated from?

☐ No ☐ Yes, I owned ______________ ☐ Yes, I still own ___________________

Were you involved in agriculture?

☐ Yes | ☐ No

Problem	yes	no
Educational opportunities		
Land tenure issues		
Problems with agriculture<br><br>(own production, low or decreasing productivity, weather and climate issues,…)		
Landscape / Demographic changes (erosion, deforestation, other people abandoning the village)		
Marriage related		
Finance related		
Facilities (hospital, bank, infrastructure)		
Political / Social		

Development project like dams / roads etc		
Others		

If yes, whose land did you work on?

☐ Own agricultural land ☐ Parents´ agricultural land
☐ Other family´s member´s agricultural land ☐ Other person´s agricultural land

Problems

Problems **before** migrating

Problems **after** migrating

Problem	yes	no
Educational opportunities		
Land tenure issues		
Problems with agriculture<br><br>(own production, low or decreasing productivity, weather and climate issues,…)		
Landscape / Demographic changes (erosion, deforestation, other people abandoning the village)		
Marriage related		
Finance related		
Facilities (hospital, bank, infrastructure)		
Political / Social		
Development project like dams / roads etc		
Others		

Income

How has migrating affected your household income?

☐ Positively | ☐ Negatively

Household income

Before migrating ____

After migrating ____

What was your main source of income **before** migrating to Dehradun?

What is your main source of income **after** migrating to Dehradun?

Conditions of the rural area

How easy do you think it is to work in a non-agricultural sector in your village?

☐ Impossible ☐ Difficult ☐ Normal ☐ Easy

Have you ever visited your village after migrating?

☐ Yes | ☐ No

If yes, for what reason?

If given the chance, would you like to go back to your native village?

☐ Yes | ☐ No

If yes, under which condition would you go back to your village?

Your view on rural-urban migration in Uttarakhand

Which, do you think, is the reason most people migrate from rural to urban areas in Uttarakhand?

What do you think was more relevant for migrating in your case?

Factors	Example	Choice
**Push**<br><br>(bad conditions in the rural area)	too low income, negatively influencing development projects like dams / roads. limited opportunities, no available potential marriage partners	
**Pull**<br><br>(attractive conditions of the urban area)	education opportunities, infrastructure, facilities like hospitals etc, enjoyment, security	

What is the major attraction of the immigrants to this area?

What is the major attraction of the immigrants to this area?

Is there anything that you are missing after migrating (social / cultural / environmental etc)?

☐ Yes | ☐ No

If yes: please specify.

Do you know other persons who migrated from rural to urban areas?

☐ Yes | ☐ No

If yes, could you tell us their names and a contact?

Thank you for taking the time to fill out our survey. Your input is greatly appreciated.
