# Supplementary material for: Is the push-pull paradigm useful to explain rural-urban migration? A case study in Uttarakhand, India
Source: PLoS One. 2019 Apr 2;14(4):e0214511. doi: 10.1371/journal.pone.0214511 (PMC6445429; doi:10.1371/journal.pone.0214511)
Supplement: S2 File — (PDF) [file pone.0214511.s002.pdf]

Information Sheet for Participant

Information Sheet Content for Participant

I am a Master Student associated to the Indo-German research unit FOR 2432. That project is conducted by the *German Universities of Göttingen and Kassel* and in close collaboration with a partner consortium in *India*. While FOR2432 addresses agricultural transition processes in the rural-urban interface of the emerging megacity Bangalore, my interest is in rural-urban migration in contrasting settings, such as Dehradun.

What does it mean to be a participant in this research?
Your answers will help to understand how rural-urban migration may be motivated and how it relates to agriculture and the environment. Please answer truthfully and as precisely as possible. It does not demand any costs for the participant but some hours of your time.

What are your rights as a participant in this research?
You have the right to refuse to answer any question you feel uncomfortable with without any explanation. You may ask for further information about the research and clarify the use of any answer you are about to give.

What will the information be used for?
I will collect answers of ca. 100 participants, analyse them statistically, and interpret them in context with other research findings, for example on land use changes. Only my supervisors and researchers of the FOR 2432 research group will have access to your data. Your personal data will be anonymized, and confidentiality is ensured throughout. This research may also be used for publications in academic journals; however, you will remain anonymous in any case.

Participants' oral consent:
'I agree to participate in this interview and I understand that:

i. All data collected will be kept secure, either under lock or on a computer database accessible by password only.
ii. My identity will remain confidential and anonymous.
iii. I have the right to refuse to answer questions.
iv. Information will be used for a MSc Thesis, as well as for publications, discussion papers and articles in academic journals.'

Thank you for participating in the survey!
